# Criteria to define innovation in the field of medical devices: a Delphi approach

**DOI:** 10.3205/hta000138

**Published:** 2024-02-28

**Authors:** Daniela D’Angela, Antonio Migliore, Iñaki Gutiérrez-Ibarluzea, Barbara Polistena, Federico Spandonaro

**Affiliations:** 1 C.R.E.A. Sanità (Centre for Applied Economic Research in Healthcare), Rome, Italy; 2 University of Rome Tor Vergata, Rome, Italy; 3 Basque Foundation for Health Innovation and Research (BIOEF), Barakaldo, Spain; 4 Osteba, Basque Office for HTA, Barakaldo, Spain

**Keywords:** medical devices, innovation, Delphi, Italy

## Abstract

Defining innovation in the field of medical devices can be extremely challenging due to the peculiarity of the products within this class. Short life-cycle, incrementality, learning curve effect, impact of the organizational setting, uncertainty of effect and level of evidence are only some of these aspects. A clear set of criteria to define innovation would be of paramount relevance in this field. Twelve criteria to define innovation were proposed to a multistakeholder panel within a consensus process. A Delphi method on two rounds was used to reach consensus. In total, 53 of the 93 (47%) invited panelists responded to the first round of the survey. Among them, 51 (96%) completed also the second round. At the first round, consensus was reached for four of the 12 proposed criteria. Three of the remaining eight criteria reached consensus at the second round. It was not possible to reach consensus for the remaining five criteria. The criteria that collected the highest scores (close to 100%) were from the clinical impact domain, namely the ability of the technology to offer significant advantages over existing alternatives in terms of improving relevant clinical outcomes, and the ability to address an unmet need defined in terms of unavailability of diagnosis/treatment alternatives. High levels of consensus (about 80%) were registered on criteria belonging to non-clinical domains of analysis and, in particular, the ability of the technology to introduce organizational benefits, and the ability of the technology to bring cost reduction providing the same clinical benefit of current alternatives.

## Introduction

Defining innovation in the field of medical devices can be extremely challenging due to the peculiarity of the products within this class. Short life-cycle, learning curve effect, impact of the organizational setting on the performance, and level of evidence are only some of the specific aspects entangled in the assessment of a medical device as those are related to ascertaining their value. A clear set of criteria to define innovation would be of paramount relevance in this field. In their pioneering review, Ciani et al. [1] systematically searched for definitions of innovation in relation to medical devices and classified them according to innovation management and economics theory. They concluded that the innovative value of medical devices cannot ignore its multidimensional and perceptive nature and should not be restricted to the sole therapeutic added value under the healthcare policy-makers perspective. More recently, Syeed et al. [2] published a systematic review to describe the characteristics of healthcare innovation within the field of both treatment and services. They were able to describe eight attributes of innovation: novelty, step change, substantial benefits, an improvement over existing technologies, convenience and/or adherence, added value, acceptable cost, and uncounted benefits. More recently, Rejon-Parrilla et al. [3] in an analysis of how innovation is defined among HTA organizations and countries found differences among countries and in how they operationalize the concept. However, they did not explore differences among health technologies. Furthermore, the way in which value is defined and by whom is crucial when determining what innovation means and could differ among stakeholders [4] and context [3]. These are the reasons why reaching to a consensus among different stakeholders on how to measure value [5] and what innovation means is so important. In addition to that, defining criteria that could be commonly accepted, despite that each of the individual criterion could be differently scored or serve to prioritize depending on stakeholders’ views and context, is of paramount significance. The present study has a focus on the Italian context where there are no mechanisms or frameworks in place to acknowledge innovation in the field of medical devices. New medical devices enter the health care system, in many occasions, from the “side door”, by direct offer to the final decision-makers (clinicians and hospital managers) and rely on the presence of the CE mark to justify their use in clinical settings [6], [7], although the technology could not be mature enough to fit for purpose in real practice. That’s the case for many countries in Europe. No assessment of any kind is currently performed for the vast majority of products either at a national or regional level, except for those that the regulation currently in place requires to do so. Furthermore, no HTA analysis is performed in order to demonstrate products’ value in clinical practice.

## Methods

### Study design 

A two-round Delphi method was used to reach consensus among the panelists [8]. This method has been previously used in order to reach consensus on how to evaluate medical devices [9]. After the identification of the research problem, a comparative analysis on innovation management programs implemented in different countries was performed and a set of criteria used to acknowledge innovation to medical devices were identified by the authors. This process resulted in a list of 12 criteria. A multi-stakeholder panel received an e-mail invitation to participate in the study and completed the survey rounds (two rounds were decided a priori). All data were analyzed with descriptive statistics.

### Panel 

The panel was selected by convenience sampling and consisted of 93 individuals identified from different stakeholder categories in Italy: governmental institutions (n=25; 27%), scientific societies and foundations (n=21; 23%), healthcare management (n=17; 18%), industry (n=8; 9%), healthcare professionals (n=8; 9%), patient associations (n=8; 9%), academia (n=5; 5%), and independent HTA experts (n=1; 1%).

### Questionnaire and survey 

Based on the comparative analysis on innovation management programs implemented in different countries in Europe and United States, 12 criteria were defined and stratified into the following five dimensions (Table 1 (Tab. 1)): general (3 criteria); clinical impact (3 criteria); quality of evidence (2 criteria); organizational impact (1 criterion); economic impact (3 criteria). The first Delphi round was launched to the panel 5^th^ May 2020. Panelists used a dedicated online platform to participate and a timeline of 15 calendar days was set to provide answers. A further 15 days were granted after a reminder e-mail. The agreement was defined using a 9-point scale where scores from 1 to 3 were used to indicate a low level of importance of the criterion (i.e., not very important to acknowledge innovation), scores from 4 to 6 were used to indicate a medium level of importance and finally, scores from 7 to 9 were used to indicate a high level of importance (i.e., very important to acknowledge innovation). The cut-off for consensus was set at a minimum of 70% of the number of respondents, meaning that strong disagreement or agreement was considered reached if at least 70% of participants had assigned scores in the range 1–3 or 7–9 to that criterion, respectively [10]. Statements that had not reached the threshold, were resubmitted to the respondents in a second Delphi round without changes. Results of the first round were shared with the respondents.

## Results

**Figure 1:**
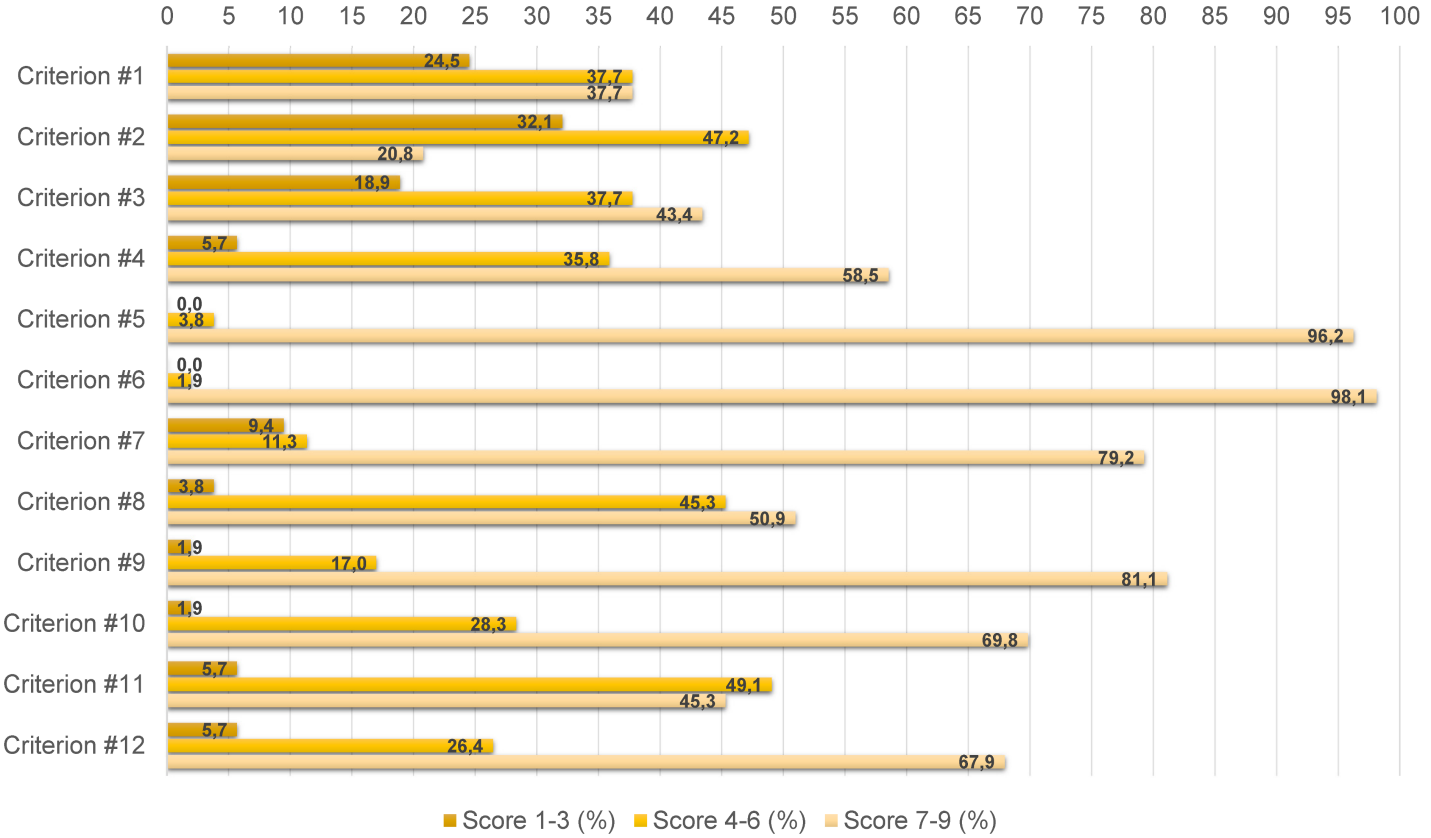
Results from the first round of the Delphi process

**Figure 2:**
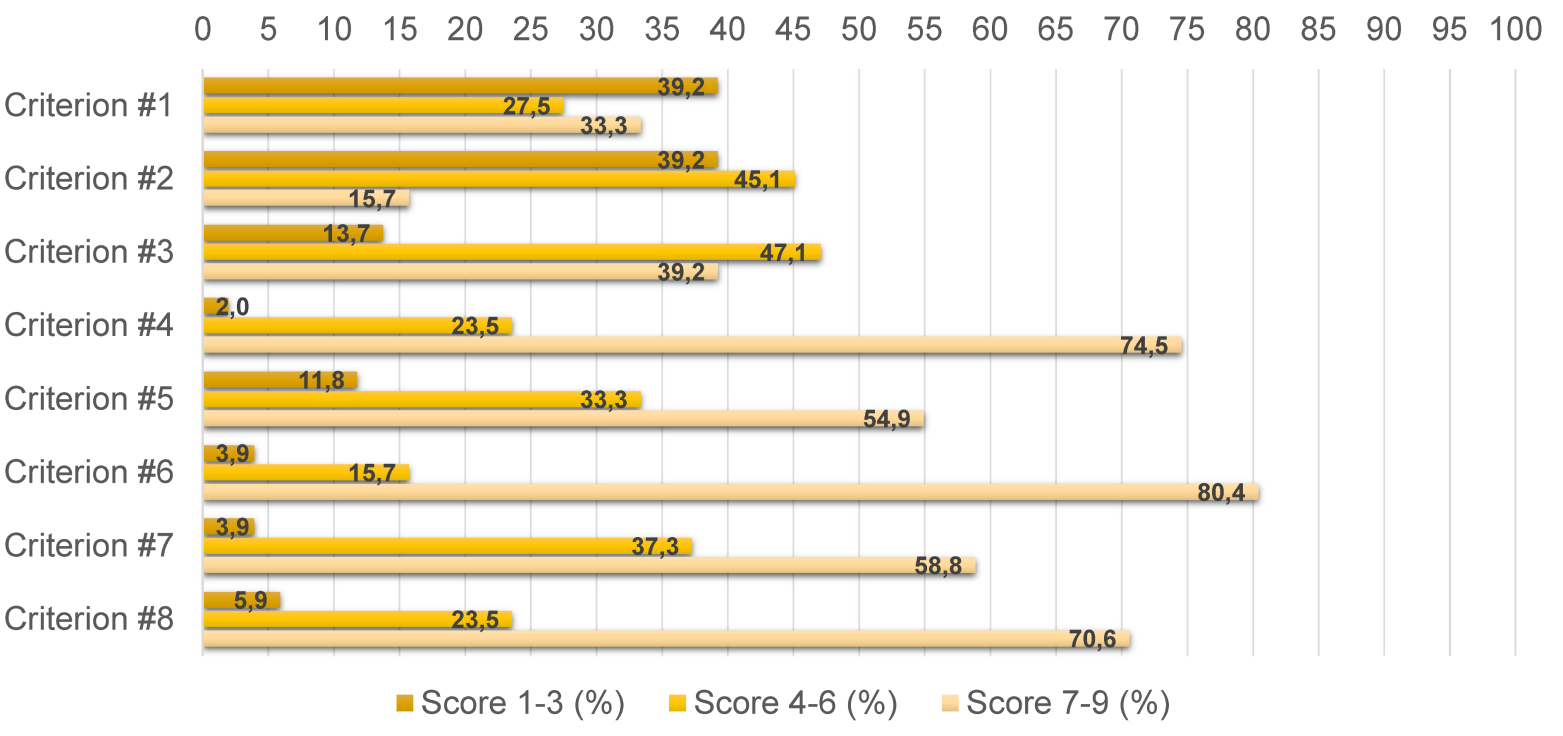
Results from the second round of the Delphi process

As per protocol, the Delphi process was concluded in two rounds. A response rate of 53% was obtained at the first round where 53 of the 93 invited individuals responded to the survey. Distribution of the different stakeholder categories between the initial panel and the responding panelists at the first round didn’t change significantly: institutions (n=12; 23%), scientific societies and foundations (n=14; 26%), healthcare management (n=7; 13%), industry (n=6; 11%), healthcare professionals (n=4; 8%), patient associations (n=6; 11%), academia (n=3; 6%), and independent HTA experts (n=1; 2%). The cut-off for agreement (i.e., 70%) was reached for 4 of the 12 proposed criteria. This is presented in Figure 1 (Fig. 1), together with the respective scores of all criteria. The remaining 8 criteria were submitted for the second round without changes and a response rate of 96% was achieved considering that 51 of the 53 responders completed the survey. Agreement was reached on further 3 criteria, presented in Figure 2 (Fig. 2) with their respective scores. At the end of the Delphi process, 7 out of the 12 criteria met the consensus of the panel and they are presented in Table 2 (Tab. 2).

## Discussion

Consensus (defined a priori with a cut-off of 70%) was achieved for 7 of the 12 criteria initially proposed; this results wasn’t reached for the remaining 5 criteria. The criteria for which the panel acknowledged a high level of importance (i.e., those receiving the highest scores) with a very strong consensus among respondents (close to 100%) were the 2 criteria from the clinical impact domain, namely criterion #6 (98% consensus at the first round) related to the ability of the device to offer significant advantages over existing alternatives in terms of improving relevant clinical outcomes and criterion #5 (96% consensus at the first round) the one on the ability of the device to respond to an unmet need defined in terms of unavailability of diagnosis/treatment alternatives for the same indication. An analogy can be made with the criteria defined by the Italian Agency for Pharmaceuticals (Agenzia Italiana del Farmaco, AIFA), for the acknowledgement of the innovative value of pharmaceutical products [9]. This is not surprising, being the panel composed by Italian stakeholders, all quite familiar with such a framework. AIFA defines “therapeutic need” as one of the three criteria and translates it as the availability of therapeutic alternatives for the condition and the associated clinical outcomes; similarly, “added therapeutic value” is defined by AIFA according to the magnitude of clinical benefit of the new treatment, measured against available therapeutic alternatives and on clinically relevant outcomes.

Consideration should also be given to the high level of consensus achieved from criteria belonging to nonclinical domains of analysis and, in particular, criterion #9 (81% consensus at the first round), related to the ability of the device to introduce organizational benefits, and criterion #10 (80% consensus at the second round), related to the ability of the device to bring a cost reduction for the same clinical benefit. These results seem to reflect an overall approach of the panel that, even considering clinical elements extremely important, tend to introduce additional systemic elements which could increase the perception of innovative value of the device, also considering the resource constraint that the healthcare system faces from the government. According to this assumption, innovation should be measured also considering the impact of the device on the organizational and economic aspects. This reflects the fact that innovation is related to value, the panel acknowledged that and, as a consequence, the innovativeness should be prioritized accordingly.

Among the other criteria that reached consensus there was criterion #7 (79% consensus at the first round), related to the quality of evidence. It emerged that the panel considers quality of evidence quite an important element, even in the context of innovative medical devices, where this issue is often debated [11]. This is not surprising, though, quality of evidence is related to the certainty we have on results and that also relates to the concept of value. It’s convenient to assume that the explicative framework provided to the panelists helped their judgement. In fact, the criterion was accompanied by a note clarifying that quality of evidence coming from secondary studies (meta-analyses, systematic reviews, HTA reports) and/or primary comparative studies (either randomized or not) should be assessed according to GRADE [12] and evidence coming from non-comparative observational studies should be assessed according to other specific validated tools. *Per se*, this note is not completely exhaustive as it doesn’t go deep into the complexity of the hierarchy of evidence, another extremely debated topic in the field of medical devices. However, it provides insight into the perceived importance of the quality of evidence to assess innovative value within this context.

Another criterion that reached consensus was criterion #4 (75% consensus at the second round), related to the severity of the condition. Again, the panelists seem to acknowledge that devices aimed to the management of lethal condition and those dealing with chronicity, should have a special attention. Again, this may represent a certain level of maturity of the system, here represented by the panel, where the vision goes beyond the device itself and projects to the society as a whole.

The criterion on the cost-effectiveness ratio, criterion #12 (71% consensus at the second round), is the one at the bottom of the list of the agreed criteria. This may not surprise considering that within the Italian context, this framework is not formally adopted and rarely used to justify decision at national or regional level.

One additional interesting result is related to the finding that none of the 12 proposed criteria was believed completely unrelated to the innovation content of a medical device. In other words, all proposed criteria were somehow relevant and there was not consensus that one or more of them could be just discarded. The highest consensus obtained on lower levels of importance was about 40 percent with respect to two of the criteria classified as general: the presence of the CE mark and the recent introduction of the device on the Italian market. By extrapolation, it could be concluded that, according to the panel, a device should not claim to be innovative only according to these two elements. This shows that the concept “new means innovative” is questioned by the panelists, both in terms of access to the European and Italian market.

It has been pointed out that the considerations around what innovation means when assessing health technologies could differ from country to country [3]. Probably this is applicable to the type of technology as the way of measuring the value and the factors related to this measurement could also differ from one health technology to another and to which extent value-based healthcare frameworks have been implemented or not [12]. The proposed final set of criteria could be the basis for discussion on which a criterion could deserve higher consideration in each context. Furthermore, it could be used as criteria for prioritization of technologies based on their innovativeness. So a double utility can be assigned to this final set. The present study has some limitations. First of all, the response rate at the first round could have been higher. However, it has been reported that response rates of Delphi studies can largely vary depending on the size and composition of the panel, the length of the questionnaire, and other factors [8]. We believe that the extremely high response rate achieved at the second round can counterbalance this drawback. Another possible limitation is the time of the study since responses were received and analyzed during 2020 and finally reported in 2023. This was due to the time required by the Ministry of Health to finally release the results for external dissemination. However, we have no reasons to believe that our findings could have changed over time as no major reforms have been implemented in terms of acknowledgment of innovation of medical devices in Italy.

## Conclusions

A Delphi method on two rounds was used to reach consensus on the criteria to define innovation in the field of medical devices among a multi-stakeholder panel of 93 individuals from governmental institutions, scientific societies, healthcare management, industry, healthcare professionals, patient associations, and academia. Response rate was acceptable (53%) at first round and very high (96%) at the second round. Consensus was reached for 7 of the 12 proposed criteria. Criteria with the largest consensus were related to the clinical domain and, in particular, to the capability of the device to add a diagnostic or treatment benefit in comparison with the existing alternatives and to respond to an unmet need. However, also criteria from non-clinical domains were believed very important to acknowledge the innovative value of a device, such as its ability to have an impact on the organization of processes and procedures and on costs. The finding of the present study can be useful for the Italian context and in those contexts where medical devices are introduced in a similar fashion. Moreover, they offer an interesting starting point for the design and establishment of a structured process to acknowledge the innovative value of a medical device not only when it enters the market but also in a preliminary phase, if the processes of early assessment of value and proactive HTA and early dialogue or early advice are in place. This represents a valuable finding especially in those countries in which innovation is a criterion considered when deciding on the inclusion of a technology in the benefit package of a healthcare system or provided in an institution such as a hospital or a different clinical setting.

## Notes

### Competing interests

The authors declare that they have no competing interests.

### Acknowledgements

Authors would like to acknowledge the Italian Ministry of Health for providing the funds to support this research. 

### Funding statement

None.

## Figures and Tables

**Table 1:**
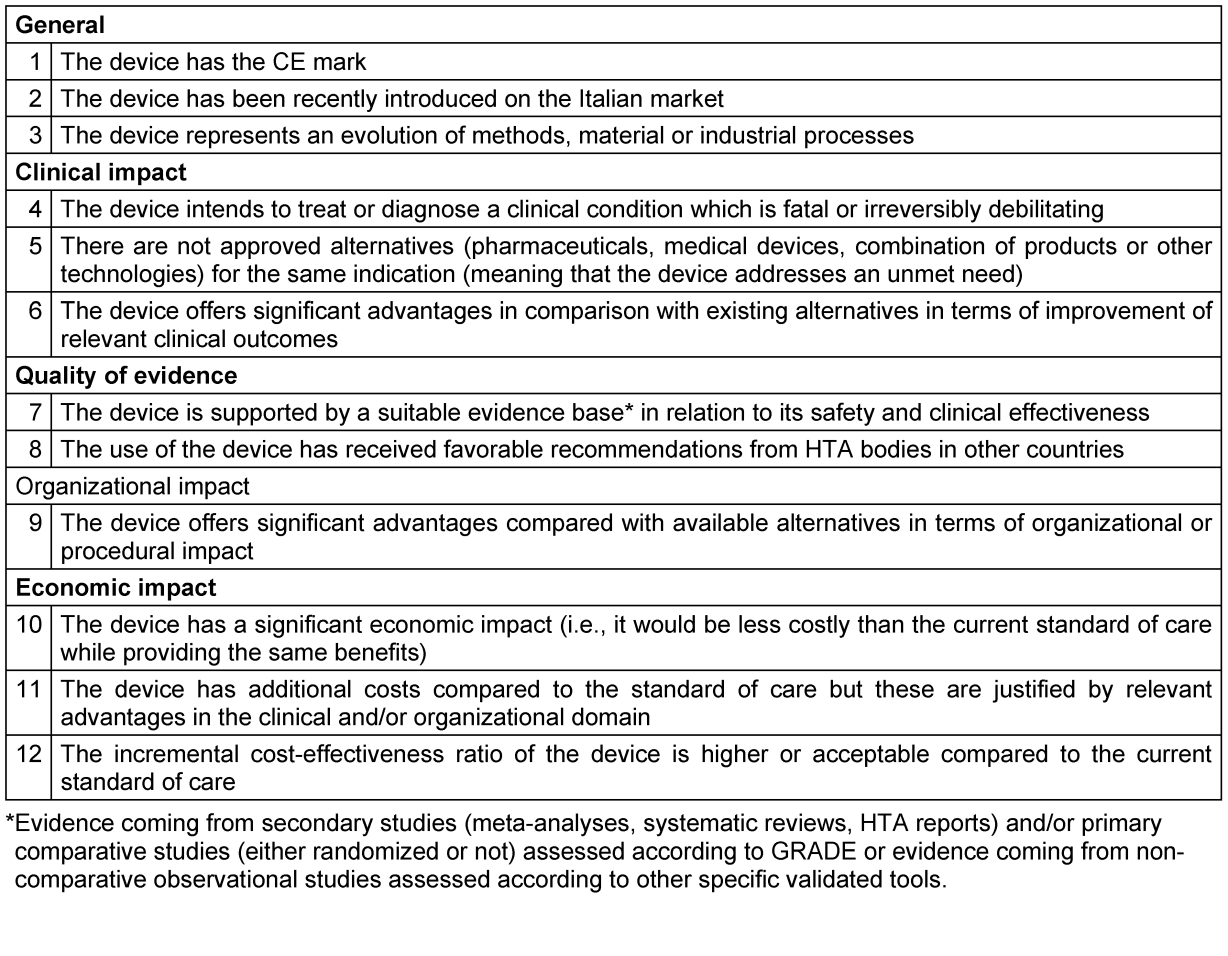
Proposed criteria to define innovation of medical devices

**Table 2:**
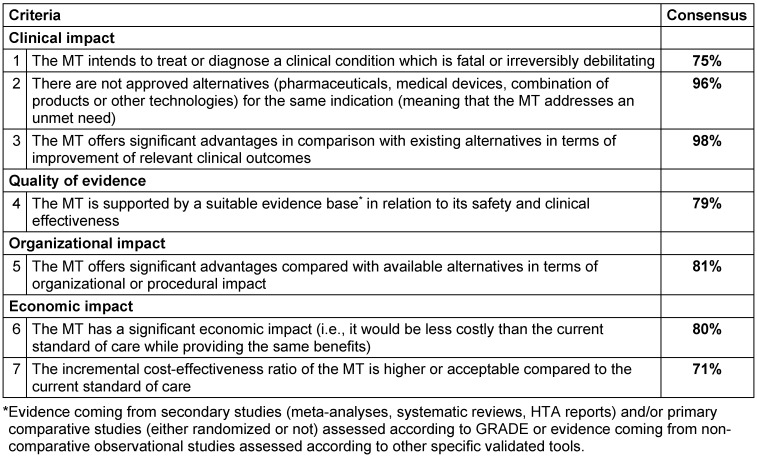
Final set of criteria to determine innovation in medical devices
